# Supplementary material for: Spatio-Temporal Variations of Volatile Metabolites as an Eco-Physiological Response of a Native Species in the Tropical Forest
Source: Plants (Basel). 2024 Sep 18;13(18):2599. doi: 10.3390/plants13182599 (PMC11435382; doi:10.3390/plants13182599)
Supplement: Supplementary file 1 [file plants-13-02599-s001.zip › plants-3060598-supplementary.pdf]

Table S1- Compounds of different organs of *Piper rivinoides* (vegetative phenological stage) from Pedra Branca State Park/ RJ.that occur simultaneously in each intersection of the Venn diagram presented in Figure 2.

Names	total	elements
Roots : Steams : Branches : Leaves	1	α–Pinene
Roots : Steams : Leaves	1	α–Muurolol
Roots : Branches : Leaves	5	δ–Amorphene; Camphene; γ–Terpinene; β–Pinene; Limonene
Roots : Steams	2	Dillapiole; Apiole
Roots : Leaves	11	α–Phellandrene; Caryophyllene oxide; Myrcene; α–Thujene; α–Cadinol; γ–Cadinene; Spathulenol; Cubenol; Sabinene; α–Cubebene; β–Phellandrene
Steams : Leaves	1	Rosifoliol
Branches : Leaves	7	γ–Elemene; *E*-Anethole; Globulol; Neryl acetate; α–Terpinene; Bicyclogermacrene; Aromadendrene
Roots	30	γ–Gurjunene; Amorpha-4,9-dien-2-ol; Safrole; Camphor; Guaiol; Germacrene D; Isoelemicin; 6-methoxy- Elemicin; Cubebol; Cadina-1,4-diene; β–Cubebene; Calacorene; Muurola-4(14),5-diene; Sibirene; Thujanol; Isoledene; Selin-11-em-4-α-ol; 3-Isopropyl-2-methoxypyrazine; Myristicin; β–Copaene; 5-hydroxy-*cis*-Calamenene; Cymene; Croweacin; Caryophllenne; γ–Asarone; Agarospirol; β–Gurjunene Guaiene; γ–Muurolene; Piperitone
Steams	1	*trans*-calamenene
Branches	14	Thujanol acetate; *allo*-aromadendrene; Myrtanol acetate; Sabinyl acetate; α–Terpineol; Linalool; Sabinene hydrate; Santolina triene; *E*-Caryophyllene; *p*-Mentha-2,4(8)-diene; Muurola-3,5-diene; *Z*-β–Ocimene; Calamenene; Verbenone
Leaves	24	Cubebol; 1,10-di-*epi*-Cubenol; α–Humulene; α–Cadinene; Cyperene; Myrcenol; *cis*-Muurola-3,5-diene; Myltayl-4(12)-ene; Sabinene hydrate; Cadina-1(6),4-diene; Viridiflorol; Cubeban-11-ol; Elemol; Mentha-2,4(8)-diene; δ–Cadinene; Calamene; 5-hydroxy-*cis*-Calamenene; Terpinyl acetate; Cymene; Caryophyllene; Linalol; α–Copaene; Muurola-4(14),5-diene

Table S2- Compounds of different phases of *Piper rivinoides* (vegetative phenological stage) from Pedra Branca State Park/ RJ that occur simultaneously in each intersection of the Venn diagram presented in Figure 2.

Names	total	elements
I: II: III: IV: V	9	α–Pinene; β–Pinene; Camphene; <d-2->Carene; Myrcene; *E*-Caryophyllene; *p*-Cymene; Limonene
I: III: IV: V	1	α–Copaene
II: III: IV: V	3	Globulol; α–Thujene; <d-3->Carene
I: II: III:	2	Dill apiole; Apiole
I: III: IV	1	Elemene
I: IV: V	1	Cubenol <1,1-di-epi>
I: II: III: IV: V	1	Cadina-1,4-diene
II: III: IV	1	Cubenol <1-epi->
III: IV: V	2	β–Phellandrene; Spathulenol
I: II:	3	*Z*-Asarone; β–Copaene; Isomyristicin
I: IV:	1	α–Terpineol
II: III	2	β–Cedrene; α–Phellandrene
II: IV	2	(*E*)-β–Ocimene; (Z)-β>Ocimene
II: V	1	β–Gurjunene
III: IV	1	bicyclogermacrene
III: V	3	Germacrene D; α–Gurjunene; β–Cubebene
IV: V	20	Muurolol; Calamenene; Caryophyllene oxide; Terpinen-4-ol; γ–Cadinene; Linalool; Selina-03,7(11)-0-diene; Sabinene hydrate; Edesmol<5-epi-7-epi-α>; Guaiadiene <6,9> NI; Mentha-2,4(8)-diene; Rosifoliol; Neryl acetate; Sabinene; Aromadendrene; E-Anethole; g-Terpinene; a-Humulene; Carotol
I	5	<6-methoxy> Elemicin; Butyl anthranilate; *E*-Carpacin; Hexadecane
II	4	Croweacin; Cumacrene; Eugenol; γ–Elemene
III	10	Libocedrol; Calamene; Tricyclene; Artemisia tiene; Heyderiol; <4-epi>Abietol; β–Ocimene; Myristicin; Niramim; Nerolidyl acetate
IV	10	Cymenene; Terpinolene; Pulegenol; Mentha-1,5-dien-8-ol; Isopulegol; Muurola-4(14),5-diene; Sabinol; Piperitol; Menth-2-em-1-ol; δ–Amorphene
V	14	Dauca-4(11),7-diene; Cadina-1(6)4-diene; *allo*-aromadendrene; α–Muurolol α–Terpinyl acetate; α–Cubebene; γ-Muurolene; Amopha-4,7(11)-diene; Cadina-1(6),4-diene; Cubenol; γ–Gurjunene; γ–Cuprenene; β-Barbatene
